# Combined antioxidant therapy with quercetin and curcumin protects against radiation-induced nephrotoxicity

**DOI:** 10.1038/s41598-026-44446-z

**Published:** 2026-04-02

**Authors:** Amr M. Abd El-Hady, Rady M. Azzoz, Saeed M. Soliman, Wafaa M. Khalil, Said A. Ali

**Affiliations:** 1 Radiology and Medical Imaging Technology Department, Faculty of Applied Health Sciences Technology, Misr University for Science and Technology (MUST), P.O. Box 77, Giza, Egypt; 2 Radiation Biology Department, National Centre for Radiation Research and Technology, P.O. Box 29, Nasr City, Egypt; 3 Biophysics Department, Faculty of Science, Cairo University, Giza, Egypt

**Keywords:** Quercetin, Curcumin, Radiation, Kidney, Rats, Cell biology, Physiology, Zoology

## Abstract

This study aimed to explore the protective effects of quercetin and curcumin against kidney damage caused by gamma radiation in male albino rats. The rats were categorized into six groups. Group I served as the untreated control. Group II received quercetin at a dose of 1.25 g/kg body weight daily by oral gavage for four weeks, followed by a nine-day break, then resumed for an additional three weeks. Group III was treated similarly with curcumin at 100 mg/kg body weight. Group IV was subjected to whole-body gamma irradiation (four doses of 2 Gy every 72 h over four weeks). Group V received both quercetin and curcumin in the same schedule as Group II, followed by gamma irradiation. Group VI was given both antioxidants after completing three consecutive weeks of radiation exposure. The findings indicated that pre-treatment with quercetin and curcumin notably improved kidney function markers (creatinine, urea, uric acid, albumin, sodium, and potassium) and antioxidant enzyme levels (CAT, SOD) compared to the irradiated group. Histological analysis and Fourier Transform Infrared (FTIR) spectroscopy confirmed structural damage in the kidneys of irradiated rats, while those pre-treated with the compounds showed largely preserved kidney tissue. These results suggest that quercetin and curcumin are effective antioxidants capable of mitigating radiation-induced kidney damage.

## Introduction

When living cells absorb ionizing radiation, it can disrupt atomic structures directly and initiate a cascade of chemical and biological responses. Indirectly, radiation interacts with water molecules, generating reactive oxygen species (ROS) that can damage cellular components such as lipids, proteins, and DNA^1^. These direct and indirect interactions trigger a range of biochemical and molecular responses that may either facilitate cellular repair mechanisms or result in lasting physiological alterations, including cell death. Furthermore, oxidative stress induced by radiation is not confined to the directly affected cells; it can extend to neighboring, non-irradiated bystander cells via intercellular signaling pathways, amplifying the overall damage within tissues^2^.

When living cells are exposed to ionizing radiation, it can directly alter atomic structures, leading to a series of chemical and biological disruptions. Indirectly, the radiation interacts with water molecules, producing reactive oxygen species (ROS) that can inflict damage on critical biomolecules such as lipids, proteins, and nucleic acids^3^. Ionizing radiation has been linked to kidney injury, commonly referred to as radiation nephropathy. It induces DNA double-strand breaks, leading to the death of various renal cell types—including endothelial, tubular, and glomerular cells—through mechanisms such as apoptosis and necrosis. Emerging evidence suggests that oxidative stress and inflammation, especially during the latent phase of the condition, play key roles in its development^4^. It is widely established that the primary lethal event in radiation exposure is the induction of DNA double-strand breaks. However, low-LET radiation, such as gamma rays, induces approximately 60–70% of this damage indirectly through the radiolysis of water and the generation of hydroxyl radicals. While antioxidants cannot repair physical DNA breaks once they occur, they may significantly reduce the total burden of damage by scavenging these reactive species before they interact with the nucleus. Quercetin is recognized for its strong antioxidant properties and its ability to neutralize free radicals^5^. It effectively scavenges superoxide anions and suppresses the activity of various enzymes responsible for superoxide production^6^. Its antioxidant potential is largely attributed to its phenolic hydroxyl groups, which enable it to donate electrons and neutralize reactive species^7^. Similarly, curcumin has been shown to reduce lipid peroxidation caused by gamma radiation and enhance the antioxidant defense mechanisms within cells^8^. As polyphenolic compounds, both quercetin and curcumin are known to alleviate intracellular oxidative stress and offer protective benefits^9^. Based on this, the present study aimed to investigate the protective mechanisms of quercetin and curcumin in mitigating radiation-induced kidney toxicity in rats.

## Materials and methods

This study was approved by the Research Ethics Committee of the National Center for Radiation Research and Technology (NCRRT), under approval number 45 A/21, chaired by Prof. Dr. Mahmoud M. Ahmed. All animal procedures strictly followed the ARRIVE guidelines and conformed to established institutional and national ethical standards. All methods were performed in accordance with the relevant guidelines and regulations. Male Sprague Dawley albino rats (120–150 g) were obtained from the NCRRT breeding facility in Nasr City, Cairo, Egypt. Before the experiment, the rats were acclimatized for one week in standard metal cages. The animals were housed in a controlled environment with a temperature maintained at 22 ± 2 °C, relative humidity of 50 ± 5%, and a 12-hour light/dark cycle. Throughout the study, they were provided with a nutritionally complete pellet diet and had unrestricted access to clean drinking water.

### Radiation exposure

Gamma irradiation procedures were carried out at the NCRRT facility in Nasr City, Cairo, Egypt. A Gamma Cell-40 irradiator (Cesium-137 source) was used to deliver radiation at a dose rate of 0.61 Gy per minute. The experimental design involved a total whole-body radiation dose of 8 Gy, administered in four separate fractions of 2 Gy each, spaced 72 h apart.

### Preparation of Quercetin and Curcumin

Quercetin, sourced from Sigma-Aldrich (St. Louis, MO, USA), was dissolved in 1 ml of normal saline and administered orally via gavage at a dose of 1.25 g/kg body weight, following an overnight fast. Similarly, curcumin, also obtained from Sigma-Aldrich, was given by oral gavage at a dose of 100 mg/kg body weight after overnight fasting^10,11^. The selection of these specific dosages and the treatment duration was based on previously validated protocols demonstrating the synergistic protective efficacy of this combination^39^.

## Experimental design

In this study, 42 male albino rats were randomly assigned to six groups, with seven rats per group. Group I served as the control and received a saline solution without any treatment. Group II was administered quercetin orally at a dose of 1.25 g/kg daily for 28 days, paused for nine days, and then resumed for another 21 days. Group III followed the same schedule as Group II but received curcumin at 100 mg/kg instead. Group IV was exposed to whole-body gamma irradiation beginning on day 28, receiving 2 Gy every 72 h to reach a total dose of 8 Gy. Group V received both quercetin (1.25 g/kg) and curcumin (100 mg/kg) on the same schedule as Group II, followed by the irradiation protocol used in Group IV. In contrast, Group VI underwent the full radiation exposure first, after which the combined treatment with quercetin and curcumin was administered daily for 21 days. The animals were sacrificed 14 days after the cessation of radiation exposure. This specific endpoint was selected to evaluate the acute phase of radiation toxicity rather than the chronic phase of radiation nephropathy.

### Sample preparation

At the end of the exposure period, animals were euthanized under deep anesthesia induced by an intraperitoneal injection of thiopental sodium at a dose of 50 mg/kg. Following confirmation of a surgical plane of anesthesia, indicated by the absence of reflexes, euthanasia was carried out by cervical dislocation^12^. Blood was collected from each rat via cardiac puncture using a sterile syringe. The samples were then transferred into heparinized tubes for biochemical evaluation. Plasma was separated by centrifugation at 1000xg for 10 min.

### Biochemical parameters

All biochemical assays were performed using commercial kits that were purchased from Biodiagnostic Trade Co. (Dokki, Egypt). Absorbance readings were taken using a UV-Vis Spectrophotometer model UV-1800 manufactured by Shimadzu (Japan). Blood superoxide dismutase (SOD) activity was determined according to a previously established method^13^, while catalase (CAT) activity was measured following a standard protocol^14^. Albumin levels were quantified using the Biuret reaction method^15^. Serum urea and creatinine concentrations were assessed using colorimetric methods^16,17^, and serum uric acid levels were evaluated using an enzymatic assay^18^. Sodium (Na⁺) and potassium (K⁺) ion concentrations were determined using an ion-selective technique^19^.

### Histological methods

For histopathological evaluation, small kidney tissue samples were fixed in 10% neutral-buffered formalin for 48 h. Tissues were dehydrated in a graded ethanol series, cleared in xylene, and embedded in paraffin to prepare tissue blocks. Sections of 5 μm thickness were cut, mounted on glass slides, and stained with hematoxylin and eosin (H&E) following a standard protocol^20^. Photomicrographs were captured using an Olympus^®^ digital camera (SC100; 10.6 MP; https://www.olympus-lifescience.com) mounted on an Olympus^®^ light microscope (CX31, Japan) equipped with 4×, 10×, 40×, and 100× objective lenses. Digital image quantification and analysis were conducted using ImageJ (Version 1.54d; https://imagej.net). All procedures were conducted at the Faculty of Medicine, Tanta University, Egypt.

### FTIR spectroscopy

The Fourier-transform infrared (FTIR) spectra of the kidney samples were obtained using a Jasco FTIR 460 Plus spectrometer (Japan). For sample preparation, finely ground potassium bromide (KBr) pellets were meticulously prepared by thoroughly mixing each sample with KBr powder. Spectra were recorded in the mid-infrared range (400–4000 cm⁻¹) with a scan speed of 2 mm/s and a resolution of 4 cm⁻¹ at room temperature. Peak bandwidths were measured at 50% of their maximum height. All analyses were conducted at the Micro-Analytical Center, Cairo University, Egypt.

### Statistical analysis

The data were analyzed using the Statistical Package for the Social Sciences software (IBM SPSS Statistics, Version 20.0; https://www.ibm.com/products/spss-statistics). A one-way analysis of variance (ANOVA) was performed to evaluate differences among groups. Results were expressed as mean ± standard deviation (SD). Statistical significance was defined as *p* ≤ 0.05, while *p* < 0.01 was considered highly significant, and *p* < 0.001 indicated very highly significant differences.

## Results

### Biochemical findings

#### Enzyme activities of blood catalase (CAT) and superoxide dismutase (SOD)

**Fig. 1:**
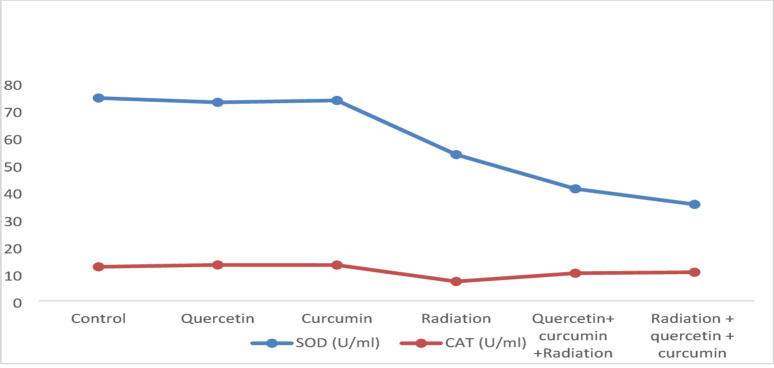
Effects of quercetin and curcumin on blood catalase (CAT) and superoxide dismutase (SOD) activities in various rat groups.

The findings showed that giving Qur or Cur orally for 28 consecutive days did not lead to any significant changes in plasma CAT and SOD enzyme activity when compared to the untreated control group. However, rats that received repeated doses of γ-irradiation experienced a notable drop-in SOD activity after 14 days (*P* < 0.05), along with a highly significant reduction in plasma CAT activity (*P* < 0.01) relative to control levels. Administering Qur and Cur before irradiation resulted in a significant reduction in SOD activity (*P* < 0.05), while giving them after irradiation caused an even more pronounced decrease (*P* < 0.01). On the other hand, both pre- and post-irradiation treatments significantly boosted CAT activity (*P* < 0.01) when compared to the respective irradiated groups. The data supporting these findings are presented in Fig. 1 and detailed in Table 1.

**Table 1:** Effects of quercetin and curcumin on catalase (CAT) and superoxide dismutase (SOD) activities in various rat groups.

Animal Group	SOD (U/ml)	CAT (U/ml)
Control	74.50 ± 2.17	12.50 ± 0.19
Quercetin % of changes from control P vs. Control	72.91 ± 2.13 −2.13% (*P* > 0.05)	13.19 ± 0.11 5.52% (*P* > 0.05)
Curcumin % of changes from control P vs. Control	73.61 ± 2.14 − 1.19% (*P* > 0.05)	13.14 ± 0.12 5.12% (*P* > 0.05)
Radiation % of changes from control P vs. Control	53.71 ± 2.51 −27.90% (*P* < 0.05)	7.11 ± 0.08 − 43.12% (*P* < 0.01)
Quercetin + curcumin +Radiation % of change from radiation P vs. radiation	41.15 ± 2.27 − 23.38% (*P* < 0.05)	10.15 ± 0.12 42.75% (*P* < 0.01)
Radiation + quercetin + curcumin % of change from radiation P vs. radiation	35.43 ± 2.31 −34.03% (*P* < 0.01)	10.51 ± 0.13 47.81% (*P* < 0.01)

Values are represented as mean ± SEM (standard error of the mean).

### Kidney function results

**Fig. 2:**
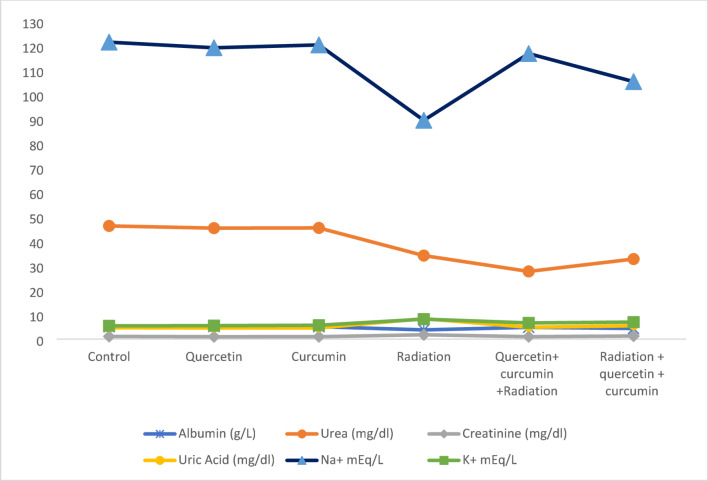
Effects of quercetin and curcumin on albumin, urea, creatinine, uric acid, sodium, and potassium levels across the various rat groups.

Oral administration of Qur or Cur for 28 consecutive days did not produce any significant changes in blood levels of creatinine, urea, uric acid, albumin, sodium (Na), or potassium (K). In contrast, rats subjected to whole-body γ-irradiation showed significantly lower levels of albumin, urea, and sodium (*P* < 0.05) fourteen days after exposure, along with a highly significant increase in creatinine, uric acid, and potassium (K) levels (*P* < 0.01). These biochemical alterations observed among the different experimental groups are presented in Fig. 2 and Table 2. Rats that received Qur and Cur before irradiation showed a significant rise in albumin levels (*P* < 0.05), a highly significant decrease in creatinine and uric acid levels (*P* < 0.01), and a highly significant increase in sodium levels (*P* < 0.01), while changes in urea and potassium levels were not statistically significant. In the post-irradiation treatment group, rats exhibited non-significant increases in albumin and sodium, non-significant decreases in urea and potassium, a significant drop in creatinine (*P* < 0.05), and a highly significant reduction in uric acid (*P* < 0.01).

**Table 2:** Effects of quercetin and curcumin on the levels of albumin, urea, creatinine, uric acid, sodium, and potassium in different groups of rats.

Animal Group	Albumin (g/L)	Urea (mg/dl)	Creatinine (mg/dl)	Uric Acid (mg/dl)	Na^+^mEq/L	K^+^ (mEq/L)
**Control**	5.05 ± 0.29	46.31 ± 0.49	1.11 ± 0.07	4.67 ± 0.14	121.47 ± 2.3	5.45 ± 0.04
**Quercetin** **% of changes from control** **P vs. Control**	5.14 ± 0.18 1.78% (*P* > 0.05)	45.41 ± 0.38 − 1.94% (*P* > 0.05)	0.99 ± 0.03 − 10.81% (*P* > 0.05)	4.56 ± 0.13 − 2.35% (*P* > 0.05)	119.18 ± 2.1 − 1.88% (*P* > 0.05)	5.52 ± 0.05 1.28% (*P* > 0.05)
**Curcumin** **% of changes from control** **P vs. Control**	5.09 ± 0.10 0.79% (*P* > 0.05)	45.49 ± 0.40 − 1.77% (*P* > 0.05)	1.0 ± 0.02 − 9.90% (*P* > 0.05)	4.58 ± 0.19 − 1.92% (*P* > 0.05)	120.35 ± 2.19 − 0.92% (*P* > 0.05)	5.68 ± 0.07 4.22% (*P* > 0.05)
**Radiation** **% of changes from control** **P vs. Control**	3.78 ± 0.22 − 25.14% (*P* < 0.05)	34.18 ± 0.34 −26.19% (*P* < 0.05)	1.78 ± 0.12 60.36% (*P* < 0.01)	8.38 ± 0.27 79.44% (*P* < 0.01)	89.51 ± 1.76 − 26.31% (*P* < 0.05)	8.18 ± 0.15 50.09% (*P* < 0.01)
**Quercetin + curcumin +Radiation** **% of change from radiation** **P vs. radiation**	4.82 ± 0.16 27.51% (*P* < 0.05)	27.70 ± 0.30 −18.95% (*P* > 0.05)	1.01 ± 0.03 −43.25% (*P* < 0.01)	4.84 ± 0.15 −42.24% (*P* < 0.01)	116.80 ± 3.12 30.48% (*P* < 0.01)	6.63 ± 0.10 − 18.94% (*P* > 0.05)
**Radiation + quercetin + curcumin** **% of change from radiation** **P vs. radiation**	4.36 ± 0.17 15.34% (*P* > 0.05)	32.76 ± 0.27 − 4.15% (*P* > 0.05)	1.27 ± 0.07 − 28.65% (*P* < 0.05)	5.47 ± 0.19 − 34.72% (*P* < 0.01)	105.34 ± 2.19 17.68% (*P* > 0.05)	6.97 ± 0.18 − 14.79% (*P* > 0.05)

Values are represented as mean ± SEM (standard error of the mean).

### Histopathological results

**Fig. 3:**
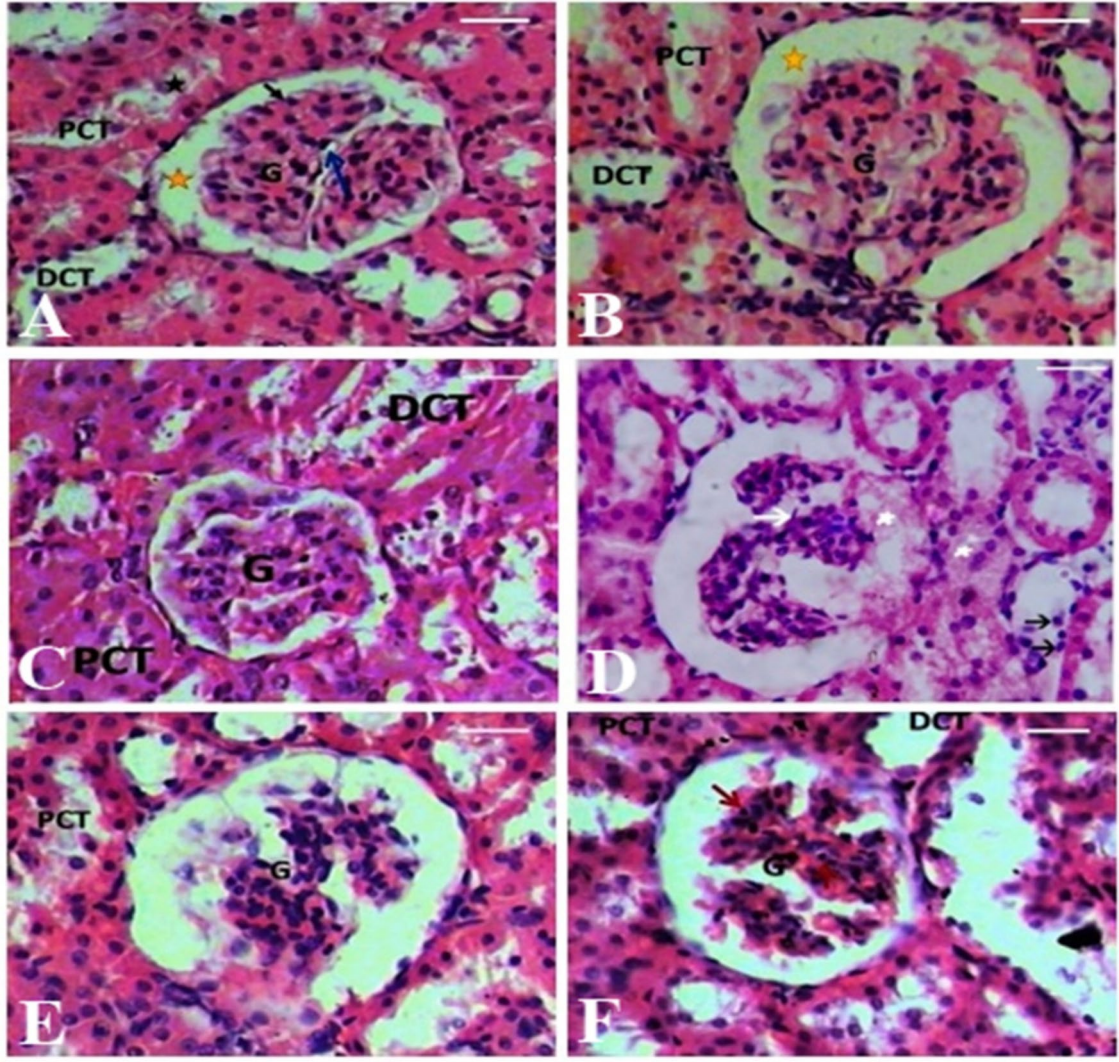
Photomicrographs of kidney sections from experimental rat groups. (**A**–**C**) Kidney cortex tissues from the control, quercetin-treated, and curcumin-treated groups exhibit normal glomerular architecture. The glomeruli contain glomerular tufts of average size (**G**), surrounded by intact endothelial cells and a clear urinary space (yellow star). Both proximal (PCT) and distal convoluted tubules (DCT) appear normal, lined with healthy epithelial cells. (**D**) Kidney cortex from the irradiated group shows marked histopathological alterations, including dilated Bowman’s capsules and hypercellular, congested glomeruli with mild architectural distortion and possible mesangial proliferation (white arrow). Tubular epithelial cells exhibit swelling and vacuolar degeneration, with occasional loss of the brush border, and some tubules contain luminal debris consistent with early tubular necrosis. Mild interstitial edema is observed, along with scattered inflammatory cell infiltrates (white star). Nuclear changes, including pyknosis and karyorrhexis, are evident (black arrow). (**E**) Kidney cortex tissue from the group treated with quercetin and curcumin prior to irradiation shows glomeruli with average-sized glomerular tufts (**G**) and a dilated urinary space (yellow star), with occasional pyknotic nuclei (black arrow). Both proximal (PCT) and distal convoluted tubules (DCT) appear structurally intact, exhibiting preserved epithelial lining, reduced tubular dilation, and minimal cellular degeneration. (**F**) Kidney cortex tissue from the group treated with quercetin and curcumin after irradiation reveals glomeruli with average-sized tufts (**G**) and widened urinary spaces (yellow star). Some endothelial cells exhibit degenerative changes, manifested as cytoplasmic vacuolation (blue arrow), while others show pyknotic nuclei (red arrow). Tubular structures show preserved morphology with reduced cellular degeneration and evidence of recovery from radiation-induced injury.

Light microscopic examination of kidney sections from the control group showed well-preserved renal architecture. The renal corpuscles appeared intact, with clear urinary spaces enclosed by thin Bowman’s capsules and healthy capillary tufts (glomeruli). Both proximal and distal convoluted tubules (PCTs and DCTs) were structurally normal; PCTs had larger diameters, prominent brush borders, and narrower lumens than DCTs (Fig. 3A). Similarly, kidney tissues from the quercetin- and curcumin-treated groups showed normal histology. The renal cortex in these groups featured intact glomeruli and tubules lined with healthy epithelial cells, indicating preserved kidney integrity (Fig. 3B and C).

Fourteen days after gamma radiation exposure, the kidney cortex from the irradiated group shows clear histopathological alterations, including marked hypercellularity, congested glomeruli, cytoplasmic vacuolation, tubular dilation, and degeneration of epithelial cells. These acute changes are consistent with the cytotoxic phase of radiation injury and provide clear evidence supporting the study’s primary claims regarding structural damage. (Fig. 3D).

These lesions are consistent with the acute phase of radiation-induced cell death and inflammation. In contrast, rats treated with quercetin and curcumin for 28 days—either before or after irradiation—demonstrated notable histological improvement. The treated groups exhibited a marked reduction in tubular necrosis and interstitial edema compared to the irradiated group. The glomeruli and tubules appeared largely restored, with only slight dilation of the urinary space in a few corpuscles (Fig. 3E and F). This suggests a protective and restorative effect of the treatments against radiation-induced kidney injury.

### FTIR spectroscopy

**Fig. 4:**
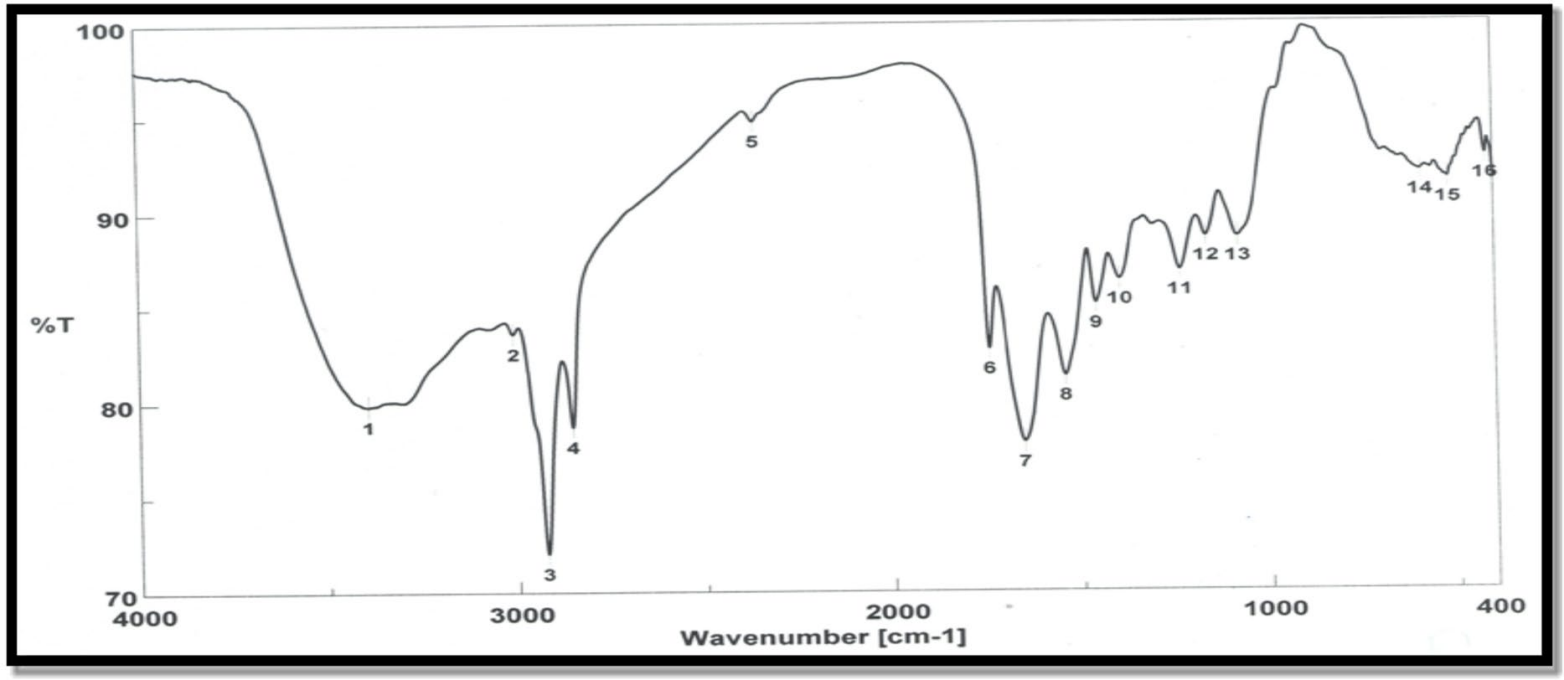
Mean FTIR spectrum of kidney tissues from the control group, recorded across the 4000–400 cm⁻¹ range.

The average FTIR spectra of control kidney cortical tissues in 4000 to 400 cm^− 1^ regions is shown in Fig. 4. The main bands are labeled in the figure, and the band assignments are given in Table 3.

**Table 3:** Characteristic band assignments of major absorbance peaks in the IR spectra of control kidney tissue within the 4000–400 cm⁻¹ range^39^.

Peak No	Wavenumber (cm^−1^)	Definition of the spectral assignment
1	3741	OH stretching
2	3289	Amide A: mainly N-H stretching of proteins
3	3006	Olefinic = CH stretching vibration: unsaturated lipids, cholesterol esters
4	2924	CH2 antisymmetric stretch: mainly lipids
5	2854	CH2 symmetric stretch: mainly lipids
6	1745	Saturated ester C = O stretch: Phospholipids, cholesterol esters
7	1651	Amide I: Protein (80% C = O stretching, 10% N-H bending, 10% C-N stretching
8	1539	Amide II: Protein (60% N-H bending, 40% C-N stretching
9	1461	CH2 bending mainly lipids, protein
10	1379	COO-symmetric stretch: fatty acids and amino acids
11	1237	PO2antisymmetric stretch nucleic acids, phospholipids
12	1164	CO-O-C antisymmetric stretching: glycogen and nucleic acids
13	1097	PO2-symmetric stretch: nucleic acids, phospholipids

The table presents the characteristic infrared (IR) absorbance bands and their spectral assignments in control kidney tissues. Peak positions and associated molecular vibrations were consistent with known functional groups, including lipids, proteins, and nucleic acids. The assignments are adapted from our previous study on liver tissue IR spectra under similar treatment conditions^39^.

**Fig. 5:**
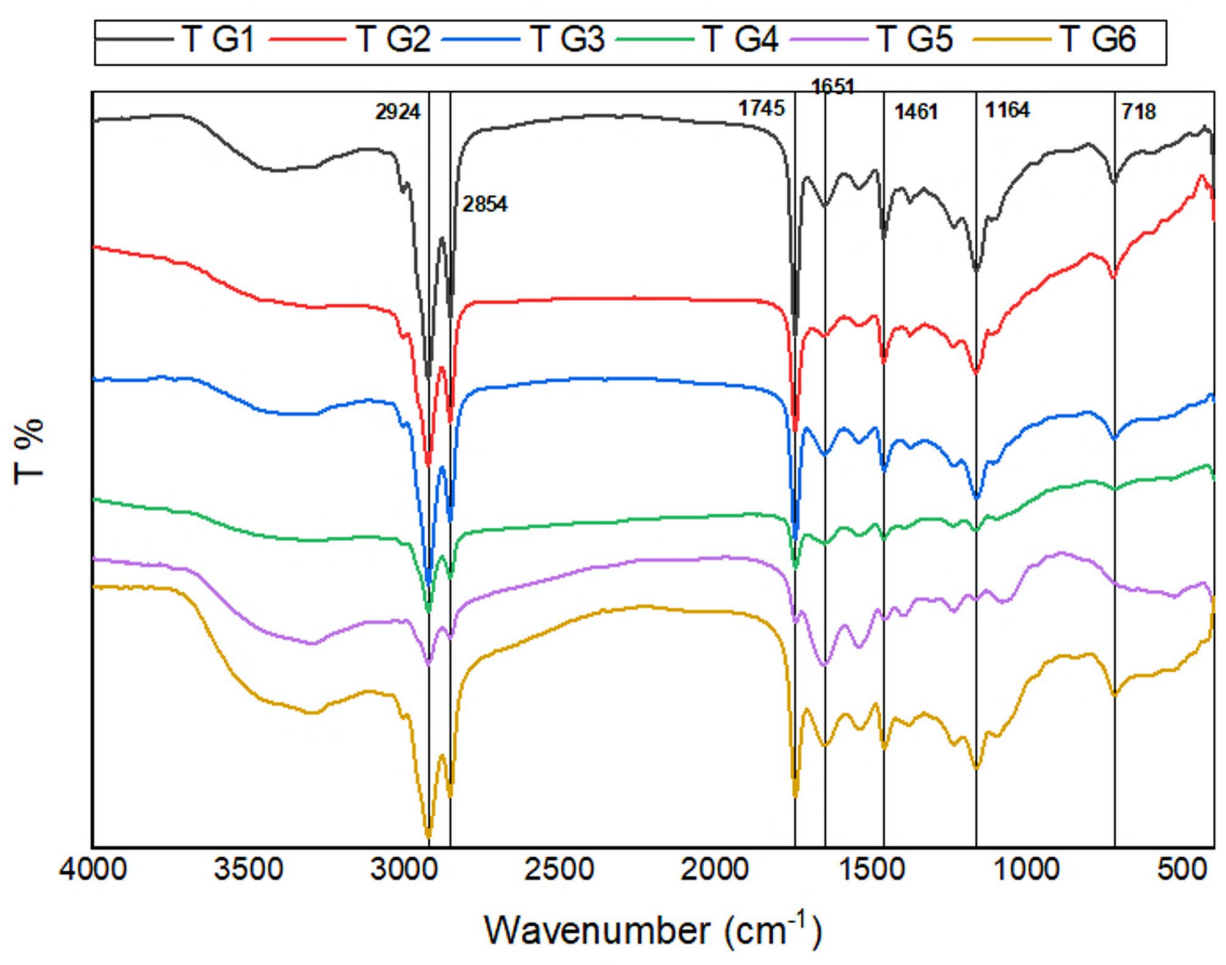
Displays the FTIR spectra of rat kidney tissues obtained after 14 days of treatment, comparing six experimental groups: control (G1), quercetin-treated (G2), curcumin-treated (G3), irradiated (G4), quercetin-curcumin combination given before irradiation (G5), and the same combination administered post-irradiation (G6). Spectral data were recorded in the mid-infrared range (4000–400 cm⁻¹), highlighting distinct molecular vibration patterns associated with each treatment group.

The average FTIR spectra of rat kidney tissues after 14 days of treatment is shown in Fig. 5, revealing minimal spectral variations across groups. In all samples, characteristic CH₂ stretching bands were observed at 2924 cm⁻¹ and 2854 cm⁻¹, with reduced intensity suggesting the presence of different amino acids. Additional peaks included a CH scissoring band at 1461 cm⁻¹ and a long-chain methyl rock at 718 cm⁻¹. A prominent, broad absorption band near 1745 cm⁻¹ appeared in all groups, though its intensity diminished in G4 and G5, likely due to C = O stretching from aliphatic ketones. The Amide I region was identified at 1651 cm⁻¹, corresponding to protein-related stretching vibrations. Meanwhile, the peak at 1164 cm⁻¹— associated with C–O–C stretching in the aromatic rings of quercetin and curcumin—showed increased intensity, indicating elevated glycogen and nucleic acid levels.

## Discussion

Ionizing radiation is extensively used in modern medicine, particularly in radiotherapy and diagnostic imaging. However, its widespread use inevitably exposes both patients and healthcare professionals to varying degrees of radiation. Kidney dysfunction caused by ionizing radiation exposure often leads to hypertension and anemia. The buildup of toxic waste metabolites further contributes to uremia, electrolyte disturbances, and chronic renal failure^13^. Gamma radiation exposure impairs renal function through several mechanisms, including oxidative deamination of amino acids, protein degradation, membrane permeability alterations, and damage to tubular epithelium and other tubulointerstitial components^14^.

In this study, the choice of a fractionated irradiation protocol (a total dose of 8 Gy administered in four 2 Gy fractions every 72 h) was strategically employed to simulate the pathophysiology of cumulative radiation-induced injury. Unlike a single bolus dose, which typically triggers acute radiation syndrome (ARS) and rapid multi-organ failure, the 72-hour interval between fractions allows for partial cellular recovery while inducing a state of chronic oxidative stress and persistent inflammatory signaling. The pathogenesis in this model is driven by the repeated production of reactive oxygen species (ROS) and the subsequent activation of pro-fibrotic and pro-inflammatory pathways. This cumulative dose rate of 0.61 Gy/min from a 137Cs source—a standard protocol validated at the NCRRT facility—is consistent with our previously established and peer-reviewed models for radiation-induced toxicity^39,44^. This approach is sufficient to overwhelm DNA repair mechanisms in renal tissues over time, leading to the observed histopathological alterations.

Fractionated irradiation protocols in the range of 6–10 Gy are widely used in experimental models to reproduce the early stages of radiation-induced nephropathy while avoiding immediate systemic toxicity. Such models allow the gradual accumulation of oxidative stress, endothelial dysfunction, and inflammatory responses that are characteristic of radiation-induced renal injury. Previous experimental studies have demonstrated that repeated sub-lethal radiation exposure promotes progressive vascular and tubular damage, which ultimately leads to functional impairment of renal filtration and electrolyte regulation^45–47^.

These pathological processes collectively disrupt renal cellular homeostasis and lead to progressive deterioration of kidney filtration and tubular reabsorption functions. These disruptions can promote electrolyte imbalances, and elevated potassium levels observed in this study may reflect radiation-induced damage to erythrocyte membranes and compromised Na⁺/K⁺-ATPase function^15^. Regarding the mechanistic action, it is crucial to distinguish between direct radiation injury and oxidative stress–mediated damage. Antioxidants do not repair the initial lethal DNA damage caused by the direct hit of gamma photons. However, the protective effects observed in this study are attributed to the mitigation of the “indirect effect” of radiation. By neutralizing the surge of ROS generated by water radiolysis, Quercetin and Curcumin minimize the secondary cascade of oxidative damage to lipids and proteins that exacerbates tissue injury in the days following exposure. This indirect oxidative pathway is considered the predominant mechanism responsible for radiation-induced cellular injury in biological tissues. The kidney is particularly vulnerable to oxidative stress because of its high metabolic activity and abundance of mitochondria, which enhances ROS generation following radiation exposure.

The present study demonstrated significant changes in key biochemical markers in irradiated rats, including altered CAT and SOD activity, alongside significant disruptions in kidney function parameters such as urea, creatinine, albumin, sodium, potassium, and uric acid. These findings are in line with previous reports highlighting gamma radiation-induced renal impairment^24–26^. The elevation of renal biomarkers such as urea and creatinine observed in the irradiated group reflects compromised glomerular filtration and indicates functional deterioration of renal tissue following radiation exposure.

CAT and SOD serve as the primary enzymatic antioxidants in renal tissue. SOD catalyzes the dismutation of superoxide radicals into hydrogen peroxide, which is subsequently broken down into water by CAT. Their combined activity plays a central role in neutralizing ROS post-radiation exposure^16^. However, gamma radiation has been shown to reduce the activity of these enzymes significantly^28,29^, potentially due to ROS-mediated enzyme inactivation and leakage from oxidatively damaged cells^30,31^. A reduction in these antioxidant defenses increases cellular susceptibility to oxidative stress, thereby accelerating lipid peroxidation, protein oxidation, and DNA damage within renal tissues.

Histopathological evaluations provided further context to these biochemical findings. Gamma radiation led to hypercellular, dilated Bowman’s capsules, congested glomeruli with mild architectural distortion, alongside tubular necrosis manifested by epithelial swelling, vacuolation, and interstitial edema. While chronic radiation nephropathy typically manifests as widespread fibrosis and glomerulosclerosis over a period of months, the histopathological changes observed in this study (at 14 days post-irradiation) are consistent with the acute cytotoxic phase of radiation injury. These early histopathological alterations likely reflect radiation-induced endothelial injury and tubular epithelial cell damage, which are considered key initiating events in radiation nephropathy. Ionizing radiation can induce microvascular dysfunction, endothelial cell senescence, and increased vascular permeability, resulting in local hypoxia and subsequent degeneration of tubular epithelial cells. These early structural disturbances ultimately contribute to progressive renal dysfunction through sustained oxidative stress and inflammatory signaling cascades^45,47^. The observed early morphological alterations substantiate the cumulative nature of the 8 Gy fractionated model. This timeline captures the transition from acute cellular detachment to early-stage organ dysfunction, providing a robust experimental window to evaluate the efficacy of Quercetin and Curcumin. This specific pathological progression has been successfully utilized in our recent investigations to evaluate radio-protective agents^39^, confirming that these early structural changes provide direct evidence of radiation-induced renal damage.

These changes are consistent with earlier reports of radiation-induced nephrotoxicity^4,32^, and may result from glomerular endothelial cell senescence, ultimately leading to structural loss^17^. Antioxidant therapy has been increasingly explored as a means of mitigating the oxidative damage induced by ionizing radiation^18^. In this context, quercetin and curcumin—two potent natural antioxidants—have demonstrated significant therapeutic potential. Quercetin neutralizes superoxide radicals and inhibits pro-oxidant enzyme activity^5,6^, while curcumin limits lipid peroxidation, prevents DNA damage, and enhances antioxidant defenses^19^. Their combined use is especially effective, as quercetin improves curcumin’s bioavailability and their synergy more efficiently reduces oxidative stress than either alone^36–39^. This synergistic interaction enhances cellular antioxidant capacity and provides stronger protection against radiation-induced oxidative injury. Histological recovery in treated rats supports these protective effects, showing significant structural improvements in both glomeruli and tubules following combined antioxidant therapy. The observed improvement in renal architecture suggests that antioxidant supplementation may attenuate the progression of radiation-induced tissue injury by stabilizing cellular membranes and preserving structural integrity.

Further support for these protective mechanisms comes from FTIR spectroscopic analysis, which showed that gamma radiation caused oxidative damage marked by lipid peroxidation and decreased CH₂ and C = O band intensities. However, the preservation of the Amide I band at 1651 cm⁻¹ may reflect partial protein repair or resistance^40,41^. Notably, the increased intensity at 1164 cm⁻¹—associated with the C–O–C bond in aromatic rings—indicates enhanced antioxidant activity from quercetin and curcumin, contributing to improved cellular defense and nucleic acid synthesis^42,43^. These spectroscopic findings further confirm the biochemical and histopathological evidence indicating that antioxidant therapy effectively reduces radiation-induced molecular damage. The reduced C = O band intensity in treated groups further supports their role in stabilizing membranes and restoring redox balance. Taken together, the biochemical, histological, and spectroscopic findings collectively support the protective role of quercetin and curcumin against radiation-induced renal oxidative injury.

Despite the clear biochemical and histopathological alterations demonstrated in this study, certain limitations should be acknowledged. The present work primarily evaluated early renal injury at 14 days following irradiation. Radiation-induced nephropathy is a progressive condition that may evolve over several months, eventually leading to fibrosis and chronic renal dysfunction. Therefore, longer observation periods and additional molecular analyses would further clarify the long-term protective effects of antioxidant therapy against chronic radiation-induced renal damage.

## Conclusion

Gamma irradiation induces significant biochemical, histopathological, and molecular alterations in renal tissues, primarily mediated by oxidative stress and disruption of antioxidant defense systems. Exposure to gamma radiation resulted in marked impairment of kidney function, as evidenced by altered biochemical markers and decreased activity of key antioxidant enzymes, including CAT and SOD. Histopathological evaluation confirmed structural damage in renal corpuscles and tubular components, reflecting early-stage radiation nephropathy.

Administration of quercetin and curcumin significantly mitigated these alterations, restoring antioxidant enzyme activity, improving biochemical parameters, and promoting recovery of renal tissue architecture. FTIR spectroscopic analysis further supported these protective effects by demonstrating reduced lipid peroxidation and improved molecular stability in treated groups.

Collectively, the integrated biochemical, histopathological, and spectroscopic evidence demonstrates a protective effect of quercetin and curcumin against gamma radiation–induced renal oxidative injury. These conclusions are directly supported by the experimental data, without overinterpretation, and provide strong evidence for the potential of combined antioxidant strategies to protect renal tissue from radiation-associated damage.

## Data Availability

All data generated or analysed during this study are included in this published article.

## References

[1] Dong, S. et al. Oxidative stress: A critical hint in ionizing radiation induced pyroptosis. *Radiat. Med. Prot.* **1**, 179–185. 10.1016/j.radmp.2020.10.001 (2020).

[2] Buonanno, M., Gonon, G., Pandey, B. N. & Azzam, E. I. The intercellular communications mediating radiation-induced bystander effects and their relevance to environmental, occupational, and therapeutic exposures. *Int. J. Radiat. Biol.* **99**, 964–982. 10.1080/09553002.2022.2078006 (2023). 35559659 10.1080/09553002.2022.2078006 PMC9809126

[3] Reisz, J. A., Bansal, N., Qian, J., Zhao, W. & Furdui, C. M. Effects of ionizing radiation on biological molecules—Mechanisms of damage and emerging methods of detection. *Antioxid. Redox Signal.* **21**, 260–292. 10.1089/ars.2013.5489 (2014). 24382094 10.1089/ars.2013.5489 PMC4060780

[4] Klaus, R., Niyazi, M. & Lange-Sperandio, B. Radiation-induced kidney toxicity: Molecular and cellular pathogenesis. *Radiat. Oncol.* **16**, 1–11. 10.1186/s13014-021-01764-y (2021). 33632272 10.1186/s13014-021-01764-y PMC7905925

[5] Cho, J. Y., Kim, I. S., Jang, Y. H., Kim, A. R. & Lee, S. R. Protective effect of quercetin, a natural flavonoid, against neuronal damage after transient global cerebral ischemia. *Neurosci. Lett.* **404**, 330–335. 10.1016/j.neulet.2006.06.010 (2006). 16806698 10.1016/j.neulet.2006.06.010

[6] Zhang, J. Y. et al. Combinational treatment of curcumin and quercetin against gastric cancer MGC-803 cells in vitro. *Molecules* **20**, 11524–11534. 10.3390/molecules200611524 (2015). 26111180 10.3390/molecules200611524 PMC6272649

[7] Pu, F. et al. Neuroprotective effects of quercetin and rutin on spatial memory impairment in an 8-arm radial maze task and neuronal death induced by repeated cerebral ischemia in rats. *J. Pharmacol. Sci.* **104**, 329–334. 10.1254/jphs.fp0070247 (2007). 17666865 10.1254/jphs.fp0070247

[8] Srinivasan, M., Sudheer, A. R., Rajasekaran, K. N. & Menon, V. P. Effect of curcumin analog on γ-radiation-induced cellular changes in primary culture of isolated rat hepatocytes in vitro. *Chem. Biol. Interact.* **176**, 1–8. 10.1016/j.cbi.2008.03.006 (2008). 18597748 10.1016/j.cbi.2008.03.006

[9] Zhao, Y. et al. The beneficial effects of quercetin, curcumin, and resveratrol in obesity. *Oxid. Med. Cell. Longev.* **2017**, 1459497. 10.1155/2017/1459497 (2017). 29138673 10.1155/2017/1459497 PMC5613708

[10] Akpolat, M., Kanter, M. & Uzal, M. C. Protective effects of curcumin against gamma radiation-induced ileal mucosal damage. *Arch. Toxicol.* **83**, 609–617. 10.1007/s00204-008-0352-4 (2009). 18754102 10.1007/s00204-008-0352-4 PMC2695547

[11] Abdel-Magied, N. & Elkady, A. A. Possible curative role of curcumin and silymarin against nephrotoxicity induced by gamma-rays in rats. *Exp. Mol. Pathol.* **111**, 104299. 10.1016/j.yexmp.2019.104299 (2019). 31442446 10.1016/j.yexmp.2019.104299

[12] Abdel-Sattar, E. et al. Pharmacological action of a pregnane glycoside, russelioside B, in dietary obese rats: Impact on weight gain and energy expenditure. *Front. Pharmacol.* **9**, 990. 10.3389/fphar.2018.00990 (2018). 30214412 10.3389/fphar.2018.00990 PMC6125411

[13] Minami, M. & Yoshikawa, H. A simplified assay method of superoxide dismutase activity for clinical use. *Clin. Chim. Acta*. **92**, 337–342. 10.1016/0009-8981(79)90211-0 (1979). 436274 10.1016/0009-8981(79)90211-0

[14] Aebi, H. Catalase in vitro. *Methods Enzymol.* **105**, 121–126. 10.1016/s0076-6879(84)05016-3 (1984). 6727660 10.1016/s0076-6879(84)05016-3

[15] Doumas, B. T., Watson, W. A. & Biggs, H. G. Albumin standards and the measurement of serum albumin with bromcresol green. *Clin. Chim. Acta* **31**, 87–96. 10.1016/0009-8981(71)90365-2 (1971). 5544065 10.1016/0009-8981(71)90365-2

[16] Fawcett, J. K. & Scott, J. E. A rapid and precise method for the determination of urea. *J. Clin. Pathol.* **13**, 156–159. 10.1136/jcp.13.2.156 (1960). 13821779 10.1136/jcp.13.2.156 PMC480024

[17] Schirmeister, J., Willmann, H. & Kiefer, H. Plasma creatinine as rough indicator of renal function. *Dtsch. Med. Wochenschr.* **89**, 1018–1023 (1964). 14135304 10.1055/s-0028-1111251

[18] Watts, R. W. E. Determination of uric acid in blood and in urine. *Ann. Clin. Biochem.* **11**, 103–111. 10.1177/000456327401100139 (1974). 4608659 10.1177/000456327401100139

[19] Solsky, R. L. & Rechnitz, G. A. Ion-selective electrodes in biomedical analysis. *Crit. Rev. Anal. Chem.* **14**, 1–52. 10.1080/10408348208542756 (1982).

[20] Bancroft, J. D. & Layton, C. The hematoxylin and eosin In (eds Suvarna, S. K. et al.) (2013).

[21] Webster, A. C., Nagler, E. V., Morton, R. L. & Masson, P. Chronic kidney disease. *Lancet* **389**, 1238–1252. 10.1016/S0140-6736(16)32064-5 (2017). 27887750 10.1016/S0140-6736(16)32064-5

[22] Robbins, M. E., O’Malley, Y., Zhao, W., Davis, C. S. & Bonsib, S. M. The role of the tubulointerstitium in radiation-induced renal fibrosis. *Radiat. Res.* **155**, 481–489. 10.1667/0033-7587(2001)155[0481:trotti]2.0.co;2 (2001). 11182800 10.1667/0033-7587(2001)155[0481:trotti]2.0.co;2

[23] Moreira, O. C. et al. Effects of γ-irradiation on the membrane ATPases of human erythrocytes from transfusional blood concentrates. *Ann. Hematol.* **87**, 113–119. 10.1007/s00277-007-0378-3 (2008). 17874241 10.1007/s00277-007-0378-3

[24] Lenarczyk, M. et al. Simvastatin mitigates increases in risk factors for and the occurrence of cardiac disease following 10 Gy total body irradiation. *Pharmacol. Res. Perspect.* **3**, e00145. 10.1002/prp2.145 (2015). 26171225 10.1002/prp2.145 PMC4492761

[25] Soliman, A. F., Saif-Elnasr, M. & Fattah, S. M. A. Platelet-rich plasma ameliorates gamma radiation-induced nephrotoxicity via modulating oxidative stress and apoptosis. *Life Sci.* **219**, 238–247. 10.1016/j.lfs.2019.01.024 (2019). 30659793 10.1016/j.lfs.2019.01.024

[26] Abdel-Aziz, N. et al. The possible impact of Spirulina and Chlorella on some hematological and biochemical aspects in irradiated rats. *Arab. J. Nucl. Sci. Appl.* **55**, 130–137 (2022).

[27] Pathak, C. M. et al. Whole body exposure to low-dose gamma radiation promotes kidney antioxidant status in Balb/c mice. *J. Radiat. Res.* **48**, 113–120. 10.1269/jrr.06063 (2007). 17339750 10.1269/jrr.06063

[28] Dixit, A. K. et al. Antioxidant potential and radioprotective effect of soy isoflavone against gamma irradiation induced oxidative stress. *J. Funct. Foods*. **4**, 197–206. 10.1016/j.jff.2011.10.005 (2012).

[29] Ismail, A. F., Zaher, N. H., El-Hossary, E. M. & El-Gazzar, M. G. Modulatory effects of new curcumin analogues on gamma-irradiation–induced nephrotoxicity in rats. *Chem. Biol. Interact.* **260**, 141–153. 10.1016/j.cbi.2016.11.010 (2016). 27838230 10.1016/j.cbi.2016.11.010

[30] Hadj Abdallah, N. et al. Zinc mitigates renal ischemia-reperfusion injury in rats by modulating oxidative stress, endoplasmic reticulum stress, and autophagy. *J. Cell. Physiol.* **233**, 8677–8690. 10.1002/jcp.26747 (2018). 29761825 10.1002/jcp.26747

[31] Usoh, I. F., Akpan, E. J., Etim, E. O. & Farombi, E. O. Antioxidant actions of dried flower extracts of Hibiscus sabdariffa L. on sodium arsenite-induced oxidative stress in rats. *Pak J. Nutr.* **4**, 135–141 (2005). https://scialert.net/abstract/?doi=pjn.2005.135.141

[32] Fouad, D., Alhatem, H., Abdel-Gaber, R. & Ataya, F. Hepatotoxicity and renal toxicity induced by gamma-radiation and the modulatory protective effect of *Ficus carica* in male albino rats. *Res. Vet. Sci.* **125**, 24–35. 10.1016/j.rvsc.2019.05.010 (2019). 31125819 10.1016/j.rvsc.2019.05.010

[33] Aratani, S. et al. Radiation-induced premature cellular senescence involved in glomerular diseases in rats. *Sci. Rep.* **8**, 16812. 10.1038/s41598-018-34893-8 (2018). 30429495 10.1038/s41598-018-34893-8 PMC6235850

[34] Nuszkiewicz, J., Woźniak, A. & Szewczyk-Golec, K. Ionizing radiation as a source of oxidative stress—The protective role of melatonin and vitamin D. *Int. J. Mol. Sci.* **21**, 5804. 10.3390/ijms21165804 (2020). 32823530 10.3390/ijms21165804 PMC7460937

[35] Srinivasan, M. et al. Modulatory effects of curcumin on γ-radiation-induced cellular damage in primary culture of isolated rat hepatocytes. *Environ. Toxicol. Pharmacol.* **24**, 98–105. 10.1016/j.etap.2007.03.001 (2007). 21783796 10.1016/j.etap.2007.03.001

[36] Grill, A. E., Koniar, B. & Panyam, J. Co-delivery of natural metabolic inhibitors in a self-microemulsifying drug delivery system for improved oral bioavailability of curcumin. *Drug Deliv. Transl. Res.* **4**, 344–352. 10.1007/s13346-014-0199-6 (2014). 25422796 10.1007/s13346-014-0199-6 PMC4240011

[37] Al Anany, M. G., Kamal, A. M. & El Saied, K. Effects of curcumin and/or quercetin on nicotine-induced lung and liver toxicity in adult male albino rat. *Al-Azhar Assiut Med. J.* **13**, 93–103 (2015).

[38] Srivastava, N. S. & Srivastava, R. A. K. Curcumin and quercetin synergistically inhibit cancer cell proliferation in multiple cancer cells and modulate Wnt/β-catenin signaling and apoptotic pathways in A375 cells. *Phytomedicine* **52**, 117–128. 10.1016/j.phymed.2018.09.224 (2019). 30599890 10.1016/j.phymed.2018.09.224

[39] El-Hady, A. M. A. et al. Studies on the effect of curcumin and quercetin in the liver of male albino rats exposed to gamma irradiation. *Histochem. Cell Biol.* **162**, 299–309. 10.1007/s00418-024-02300-1 (2024). 38913116 10.1007/s00418-024-02300-1 PMC11364652

[40] Wharfe, E. S. et al. Fourier transform infrared spectroscopy as a metabolite fingerprinting tool for monitoring the phenotypic changes in complex bacterial communities capable of degrading phenol. *Environ. Microbiol.* **12**, 3253–3263. 10.1111/j.1462-2920.2010.02300.x (2010). 20649644 10.1111/j.1462-2920.2010.02300.x

[41] Bodîrlău, R., Teacă, C. A. & Spiridon, I. Preparation and characterization of composites comprising modified hardwood and wood polymers/poly(vinyl chloride). *BioResources*10.15376/biores.4.4.1285-1304 (2009).

[42] Porto, I. C. et al. Use of polyphenols as a strategy to prevent bond degradation in the dentin–resin interface. *Eur. J. Oral Sci.* **126**, 146–158. 10.1111/eos.12403 (2018). 29380895 10.1111/eos.12403

[43] Patla, S. K., Mukhopadhyay, M. & Ray, R. Ion specificity towards structure-property correlation of poly(ethylene oxide)[PEO]-NH₄I and PEO-KBr composite solid polymer electrolyte. *Ionics* **25**, 627–639. 10.1007/s11581-018-2711-3 (2019).

[44] El-Hady, A. M. A. et al. Protective effects of antioxidant therapy against radiation-induced tissue injury. *Discover Appl. Sci.* **7**, 1338. 10.1007/s42452-025-07810-8 (2025).

[45] Cohen, E. P. & Robbins, M. E. C. Radiation nephropathy. *Semin. Nephrol.* **23**, 486–499. 10.1016/s0270-9295(03)00093-7 (2003). 13680538 10.1016/s0270-9295(03)00093-7

[46] Hall, E. J. & Giaccia, A. J. *Radiobiology for the Radiologist* 8th edn. (Wolters Kluwer, 2018).

[47] Fajardo, L. F., Berthrong, M. & Anderson, R. E. *Radiation Pathology* (Oxford University Press, 2001).

